# Supplementary material for: Techno-Economic Analysis of the Multiple-Pass Ultrasonication with Mechanical Homogenization (MPUMH) Processing of Processed Carrot Discards to Puree
Source: Foods. 2022 Dec 28;12(1):157. doi: 10.3390/foods12010157 (PMC9818328; doi:10.3390/foods12010157)
Supplement: Supplementary file 1 [file foods-12-00157-s001.zip › foods-2003785-supplementary.pdf]

Supplementary Material

Table S1 Unit production cost (Carrot puree/can) weighing 150 g for (i) Base case (ii) Case 2

Description	Sale price/unit (Can$)	Base case	Case 2
Number of carrot puree cans	0.6	41,821,972.	49,550,805
Total Revenue		25,093,183.2	29,730,483
Operating cost		23,601,216.4	8,978,682.84
Unit production cost		0.56	0.18

Table S2 Comparison of the total utilities expense incurred for (i) Base case (ii) Case 2

		Base case		Case 2	
Description	Unit cost (Can$)	Annual Rate	Annual cost (Can$)	Annual Rate	Annual cost (Can$)
Std Power	0.075	2,829,546	212,215.95	3,351,725	251,379.375
Steam	12	292	3,504	346	4,152
Steam (High P)	0	0	0	0	0
Cooling Water	0.07	19,640	1,374.8	23,269	1,628.83
Chilled Water	0.4		0		0
Recovered Steam	0		0		0
Cooling air	0		0		0
Total Utilities			217,094.75		257,160.205
			0.22 M		0.26 M

*Base case (Puree prepared from peeled processed carrot discards), Case 2 (Puree prepared from processed carrot discards and carrots (50:50), without peeling)

Table S3 Comparison of the labor cost incurred for (i) Base case (ii) Case 2

	Base case	Case 2
Description	Annual cost (Can$)	Annual cost (Can$)
Operator	1,205,568	1,205,568
Unskilled labor (peeling)	14,742,000	0
Unskilled labor	174,720	174,720
Supervisor	458,640	458,640
Total Labor	16,580,928	1,838,928
	16.66 M	1.84 M

Table S4 Total operating cost for (i) Base case (ii) Case 2

Description	Base case (Cost Can$)	Case 2 (Cost Can$)
Total Raw Material	2,152,054.44	3,670,755.43

Total Utilities	217,094.75	257,160.205
Total Labor	16,580,928	1,838,928
Lab/Quaity control	2,487,139.2	275,839.2
Facility Dependent Cost	2,164,000	2,936,000
Total Operating cost	23,601,216.39	8,978,682.84

Since there are no co product credits, Total operating cost is the net operating cost

Table S5 Comprehensive details of costs, revenue and ROI, payback time

Description		Value taken from	Base case	Case 2
Gross Operating Costs (GOC)		Table A4	23,601,216.39	8,978,682.839
Total Revenue (TR)		Table A6	25,093,183.2	29,730,483
Net Income (Taxable Income)		(GOC-TR)	1,491,966.81	20,751,800.16
Net Profit	Tax (35%)		969,778.4265	13,488,670.1
Cash Flow			3,133,778.427	16,424,670.1
Total Capital Investment (TCI)		Table A5	10,095,750	13,702,500
ROI			31.04%	119.87%
Payback Time (yr)			3.2	0.83

(All prices are in Can$)

Formulae used for calculation of parameters

Net profit= (1-0.35)* Net income

Cash Flow= Net profit + Facility dependent cost $_i$

Return on Investment=Cash flow/ Total capital investment

Payback time = Total capital investment / Cash flow

Marcs Depreciation (7 Years)

Cash flow is negative for the first two years due to construction and installation of machinery

First year cash flow= 0.40*(Total fixed capital cost)+working capital

Second year cash flow = 0.6* (Total fixed capital)

Net Present Value $\mathrm{NPV} = \sum_{i=0}^{n} \frac{\mathrm{Valuei}}{(1+r)^i}$

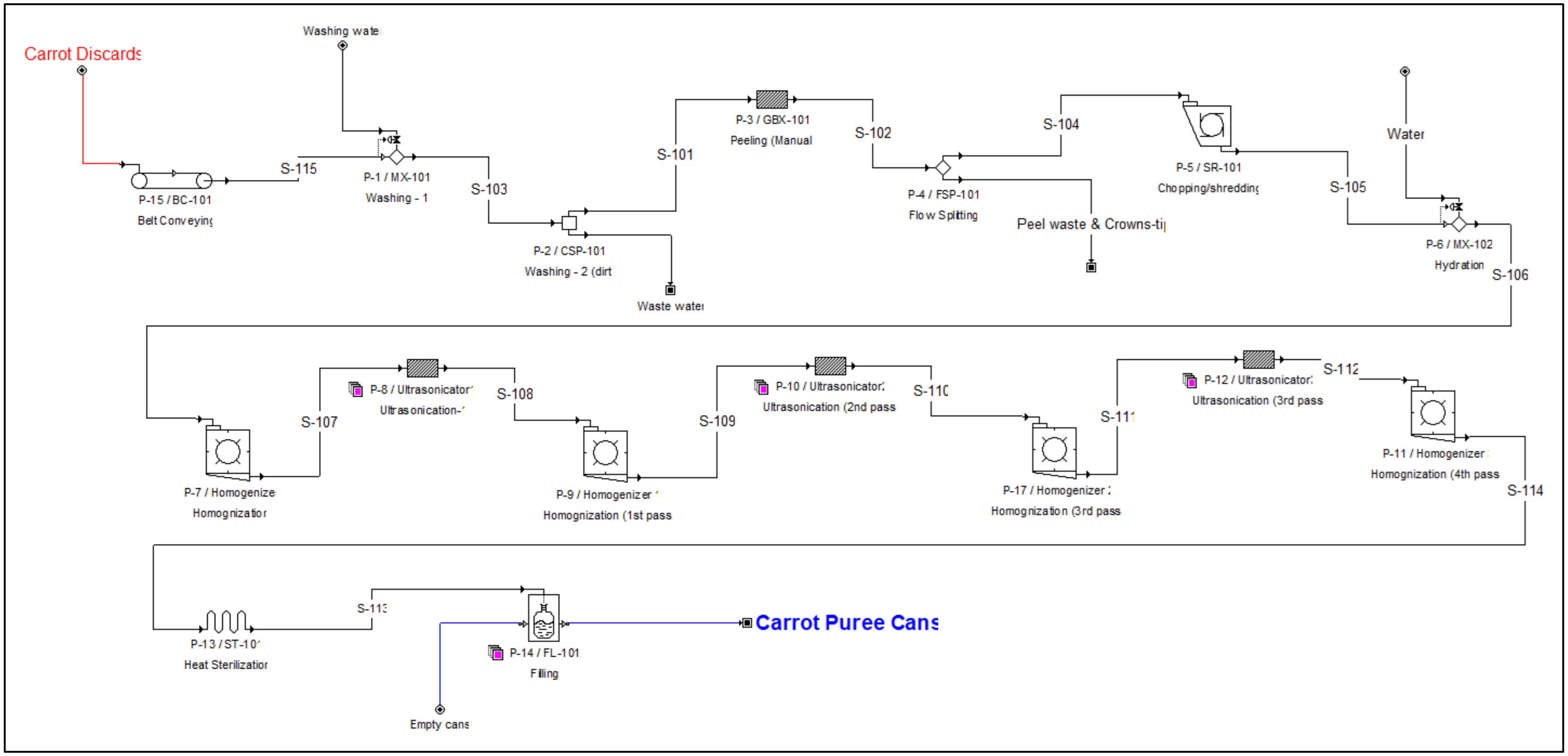

Figure S1. Flowsheet of a process model (base case) developed in SuperPro Designer V9.5.
